# Identification and evaluation of novel synovial tissue biomarkers in rheumatoid arthritis by laser scanning cytometry

**DOI:** 10.1186/ar3682

**Published:** 2012-01-17

**Authors:** Christiane Fueldner, Anja Mittag, Jens Knauer, Maria Biskop, Pierre Hepp, Roger Scholz, Ulf Wagner, Ulrich Sack, Frank Emmrich, Attila Tárnok, Joerg Lehmann

**Affiliations:** 1 Fraunhofer Institute for Cell Therapy and Immunology, Department of Cell Engineering/GLP, Perlickstr. 1, D-04103 Leipzig, Germany; 2 Department of Paediatric Cardiology, Heart Centre Leipzig GmbH, Struempellstr. 39, D-04289 Leipzig, Germany; 3 Translational Centre for Regenerative Medicine, University of Leipzig, Semmelweisstr. 14, D-04103 Leipzig, Germany; 4 Rheumatologist, Kolmstr. 2, D-04299 Leipzig, Germany; 5 Department of Trauma and Reconstructive Surgery, University Hospital Leipzig, Liebigstr. 20, D-04103 Leipzig, Germany; 6 Department of Orthopaedics, University Hospital Leipzig, Liebigstr. 20, D-04103 Leipzig, Germany; 7 Rheumatology Unit, Department of Internal Medicine 2, University Hospital Leipzig, Johannisallee 30, D-04103 Leipzig, Germany; 8 Institute of Clinical Immunology, University Hospital Leipzig, Johannisallee 30, D-04103 Leipzig, Germany

## Abstract

**Introduction:**

Suitable biomarkers are essential for therapeutic strategies in personalized medicine in terms of diagnosis as well as of prognosis. With highly specific biomarkers, it is possible, for example, to identify patients with poor prognosis, which enables early intervention and intensive treatment. The aim of this study was to identify and validate biomarkers and possible combinations for a prospective use in immunoscintigraphy, which may improve diagnosis of rheumatoid arthritis (RA) patients with consideration of inflammatory activity in the affected joints. Therefore, we tested several monoclonal antibodies (mAbs) directed against cellular-surface molecules on cells likely to be involved in the pathogenesis of RA.

**Methods:**

Synovial tissue from patients with long-standing RA (accompanied by synovitis with varying states of current activity) and patients with acute non-RA arthritis were stained for surface molecules on different cell types by using fluorochrome-labeled antibodies. Tissue analysis was done by laser scanning cytometry (LSC), and statistical evaluation, by discriminant analysis and ROC analysis.

**Results:**

CD11b, HLA-DR, CD90, and CD64 revealed significant differences between tissues from patients with RA and acute non-RA arthritis. Especially with the expression of CD64, both patient cohorts could be discriminated with high sensitivity and specificity. RA classification was improved by simultaneously investigating the expression of two or three different surface proteins, such as HLA-DR, CD90, and CD29 in the tissue. The simultaneous analysis of CD64 together with CD304 or the combination of CD11b and CD38 was suitable for the identification of RA patients with high current activity in synovitis.

**Conclusions:**

In this study, we showed that LSC is a novel reliable method in biomarker prevalidation in RA. Hence, identified mAbs *in situ *may allow their potential use in *in vivo *approaches. Moreover, we proved that biomarker-combination analysis resulted in better discrimination than did single-marker analysis. Combinations of these markers make a novel and reliable panel for the discrimination between RA and acute non-RA arthritis. In addition, further expedient combinations may be novel promising biomarker panels to identify current activity in synovitis in RA.

## Introduction

Rheumatoid arthritis (RA) is a chronic inflammatory disease characterized by infiltration of cells into the synovial tissue and progressive destruction of cartilage and bone. Cell types known to be involved in RA pathogenesis in the joint are, among others, mononuclear immune cells and fibroblasts [1].

For successful therapeutic intervention for RA with the focus on individualized medicine, it is useful to have procedures for specific and sensitive diagnosis as well as exact disease staging. It is important to identify patients with destructive disease prognosis in need of intensive treatment and to spare others from potential side effects. Therefore, tools for early and reliable diagnosis, monitoring inflammatory progress and controlling therapeutic success, are of utmost importance.

Early disease staging in RA according to American College of Rheumatology (ACR)/European League Against Rheumatism (EULAR) criteria is in addition to the enumeration of involved small and large joints based mostly on blood tests measuring the erythrocyte sedimentation rate (ESR) and levels of C-reactive protein (CRP), rheumatoid factor (RF), and anti-citrullinated protein antibodies (ACPAs) [2]. Such serologic parameters do not necessarily reflect biologic actions in the target tissue of the patient and, thus, provide only imprecise information on disease activity. Despite the great need for confirmed diagnosis in RA, no specific laboratory test is available (excellently reviewed by Nakamura [3]). However, in the last decade, monoclonal antibodies (mAbs) leading to immune-modulation of the underlying pathogenic process in RA, started a therapeutic revolution. These mAbs can be radiolabeled and applied for specific diagnostic tests. The scintigraphic detection of these radiolabeled mAbs allows direct visualization of the synovitis of RA. The combination of the assessment of disease-specific cellular biomarkers directly in the joint and noninvasive high-resolution *in vivo *imaging techniques, such as immunoscintigraphy or immuno-positron-emission tomography (PET), are suitable approaches to determine alterations in the joints and hence offer valuable tools for sensitive and specific diagnosis in RA [4-7].

This study aimed to identify appropriate biomarkers for RA intended to be further validated and envisioned to be used in immunoscintigraphy or immuno-PET. To find RA-specific biomarkers, we used synovial tissue samples from patients with a long-term course of RA and from patients with acute non-RA arthritis as a control group. Several mAbs directed against cellular-surface molecules on cells, associated with the pathogenesis of RA, including adhesion molecules, activation markers, and receptors [8-12], were tested for their potential to identify appropriate RA biomarkers by discriminating RA and acute arthritis. This was realized by quantitative tissue analysis using LSC combined with advanced statistical analysis (see Additional file 1, Figure S1 for an overall scheme of the test procedure). As a high-content screening technique, LSC is the method of choice. Although microscope based, it is unbiased and highly reproducible. Furthermore, LSC is not restricted to a few fields of view, but the whole specimen is measured [13], making this method an appropriate technique for drawing conclusions about marker expression in the tissue of origin.

## Materials and methods

### Patients and samples

The study protocol has been approved by the local Ethics Committee of the University of Leipzig. All patients gave their informed written consent.

Synovial tissue was obtained by joint surgery (hand, foot, or shoulder) from patients with RA (*n *= 17) who met the American College of Rheumatology (ACR) revised criteria for the classification of RA [14]. The control group consisted of 14 patients with trauma-induced acute arthritis (knee). Information about patients is summarized in Table 1. Histologic evaluation of RA tissue sections by a pathologist specified the local current-activity status in RA synovitis, according to the histopathologic scoring system by Stiehl [15,16]. This scoring system of RA synovitis allows the characterization of synovial tissue into type I or type II synovitis. Moreover, based on a qualitative and quantitative characterization of cell infiltrations, disease activity is differentiated. The current-activity status provides information on exudative inflammatory processes on the synovial surface (in particular, fibrin exudation and granulocyte emigration) and is graded on severity from grade 1 to 3 [15,16]. RA tissue specimens were subclassified in five patients having highly current activity (grade 2 or higher; RA(++)) or 12 patients having mild current activity (less than grade 2 (RA(+)). This subclassification does not correlate with other patient data (for example, medication).

**Table 1:** Patient information

	RA (*n *= 17)	Control (*n *= 14)
Age (years)^a^	57.4 ± 13.6	42.9 ± 10.4
Gender (male/female)	5/12	9/5
Duration of disease (years)^a^	21.4 ± 9.7	0.7 ± 1.1
Corticosteroid use (yes/no)	11/6	0/14
DMARD use (yes/no)	12/5	0/14
NSAID use (yes/no)	3/14	0/14
Biologicals use (yes/no)	3/14	0/14
RF positive (yes/no)	15/2	0/14
CRP positive (yes/no)^bc^	7/5	0/14

^a^Values represent mean ± SD; ^b^positive; elevated levels of CRP (> 10 mg/L); ^c^Five patients: no data accessible. CRP, C-reactive protein; DMARD, disease-modifying antirheumatic drug; NSAID, nonsteroidal antiinflammatory drug; RF, rheumatoid factor.

### Biomarker candidates

Molecules on cell types likely to be involved in the pathogenesis of RA (that is, adhesion molecules, activation markers, and receptors) were selected, according to the literature (Additional file 1, Table S1) and investigated with fluorescence labeling on cryosections of the RA synovium. Samples were scored as follows: absent (0), mild (1), moderate (2), or marked (3) expression. Antibodies against CD11b, CD38, CD29, CD90, HLA-DR, CD64, CD304, CD4, and CD271 provided a score > 2. These fluorochrome-labeled mAbs were used either alone or in combinations to stain serial sections of synovium for subsequent quantitative LSC analysis. For further information about antibody selection and staining, see Supplementary Information 1.

### Data acquisition

Quantitative analysis of synovial tissue was performed by using a laser scanning cytometer with the corresponding WinCyte software (CompuCyte; Westwood, MA, USA). All sections from each patient were analyzed within 24 hours after staining.

The region for scanning was set around the entire tissue section to get overall information of marker expression of the investigated material. Phantom contouring (non-cell-based analysis algorithm) was applied for analysis, as it resembles the *in vivo *situation better than a single-cell-based analysis (for details, see Supplementary Information 1).

### Quantitative tissue analysis

Because phantom contouring is a non-cell-based analysis algorithm, it is essential to distinguish between tissue and background as a first step. This was based on the scatter signal of the tissue. Only phantoms including information about underlying tissue were used for further analysis (Additional file 1, Figure S3).

Each phantom contains information on integral fluorescence intensity and MaxPixel (the brightest pixel within the phantom). The median fluorescence intensity (MFI) of all phantoms was taken as a value of the averaged marker expression in the tissue. Based on negative control measurements, unspecific background intensity was defined. MFI values were corrected by subtraction of the respective MFI-negative control value. To determine the area of tissue positive for the marker, the parameter MaxPixel was used. Based on the negative control, the threshold (< 5% positive events) for autofluorescence was set for each fluorescent dye and patient, respectively. For each analysis, the percentage of phantoms with a MaxPixel value above this threshold (affected area) was determined (see Additional file 1, Figure S4).

### Statistical analysis

For statistical evaluation of results, TANAGRA (version 1.4), an open-source data-mining software, was used [17]. MFI values and affected area of RA patients and the control group were compared with the Mann-Whitney *U *test. A Kruskal-Wallis one-way ANOVA was applied for comparison of control and the RA subgroups RA(+) and RA(++). Values were considered significantly different if *P *≤ 0.05 with *P *values ≤0.001 as highly significant. In case of significance, a Mann-Whitney *U *test was used for detailed group comparison. Significance level was adjusted by Bonferroni correction in case of multivariate analyses to *P *≤ 0.017.

The discriminatory capability of the potential biomarkers identified by single antibodies or mAb combinations was tested with linear discriminant analysis (LDA). As case numbers are limited and to abate possible overfitting of data in LDA, cross-validation (10-fold with 10 repetitions) was performed after LDA. Values for sensitivity and specificity were taken from cross-validation. Likelihood ratio was calculated as LR^+^: sensitivity/(1 - specificity).

Receiver operating characteristic (ROC) analysis was used to obtain information about the discriminatory capability and the variation of sensitivity and specificity in changing the decision threshold. AUC values > 0.85 were considered to be highly discriminative, and AUC < 0.55, to be nondiscriminative. ROC analysis was performed by using the web-based ROC analysis tool from Johns Hopkins University [18].

Graphic visualization of results was performed with SigmaPlot software (Systat, Erkrath, Germany).

## Results

The whole tissue section was scanned with LSC. Values for MFI and the affected area were obtained. Both parameters were correlated (Spearman rho, 0.663).

**Figure 1:**
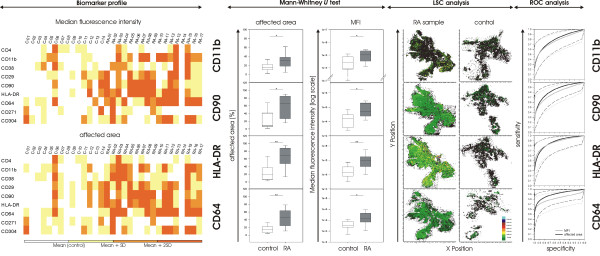
**Biomarkers for rheumatoid arthritis (RA) classification**. The biomarker profile (left) gives an overview of tested markers for each subject. For color-coding, mean of the respective control subjects +SD (+2 SD, > 2 SD) was used for each marker. CD4 and CD271 showed no difference in expression between control and RA, whereas CD64 or HLA-DR was significantly more highly expressed in RA patients. This is also apparent in color-coded tissue analysis with laser scanning cytometry (LSC). Significant differences were found for CD11b, CD90, HLA-DR, and CD64 (center, * *P *≤ 0.05; * *P *≤ 0.001). Box plots show median and 25^th^/75^th ^percentile, and whiskers, 5^th^/95^th ^percentile. Receiver operating characteristic (ROC) analysis revealed that number and distribution of labeled cells (that is, affected area) delivered most often higher sensitivity in identifying RA than did median fluorescence intensity (MFI) values. CD64 proved to be the best single discriminatory marker, with AUC = 0.8942 (right).

Although the selected mAbs provided an expression score of tagged proteins > 2, not all of the selected markers were suitable for discrimination between RA and control. In Figure 1 (left panel), values for MFI and the affected area are shown as a biomarker profile for all patients. Except for CD4 and CD271, antigens were more highly expressed in the synovium of RA patients compared with controls, enabling us to discriminate between both patient cohorts. Significantly higher values were found in RA patients regarding affected area values by CD11b, CD90, HLA-DR, CD64, CD29, and CD304 labeling (Additional file 1, Table S2, and Figure 1, center). The same applied to MFI values (except for CD29 and CD304).

Highest sensitivity values (at 90% specificity) and AUC values were obtained for HLA-DR and CD64 (Table 2, Figure 1, right panel). The highest specificity value in cross-validation for CD64 and hence, the highest likelihood ratio (Additional file 1, Table S3) makes CD64 the most promising of all tested biomarkers for a potential prognosis of RA disease.

**Table 2:** Discriminatory capability of biomarkers

		Affected area					MFI				
			
Biomarker candidate	Control vs. cohort	AUC	95% Confidence interval			**Sensit. at 90% specif**.	AUC	95% Confidence interval			**Sensit. at 90% specif**.
**CD4**	RA	0.64	0.44	-	0.84	7.35	0.57	0.35	-	0.78	9.79
	RA(+)	0.04	0.04	-	0.04	4.27	0.10	0.08	-	0.11	9.60
	RA(++)	0.68	0.43	-	0.94	19.51	0.63	0.35	-	0.90	12.75

**CD11b**	RA	0.79	0.63	-	0.95	51.82	0.76	0.60	-	0.93	51.76
	RA(+)	0.41	0.14	-	0.68	40.81	0.46	0.10	-	0.82	45.86
	**RA(++)**	**0.95**	**0.85**	**-**	**1.00**	**85.09**	**0.88**	**0.68**	**-**	**1.00**	**71.47**

**CD29**	RA	0.83	0.69	-	0.98	51.53	0.69	0.50	-	0.88	50.22
	RA(+)	0.59	0.12	-	1.00	58.59	0.62	0.00	-	1.00	61.82
	RA(++)	0.82	0.63	-	1.00	38.81	0.49	0.13	-	0.84	26.72

**CD38**	RA	0.72	0.54	-	0.91	22.23	0.70	0.50	-	0.89	17.68
	RA(+)	0.11	0.10	-	0.11	10.59	0.20	0.16	-	0.23	19.64
	**RA(++)**	**0.86**	**0.61**	**-**	**1.00**	**69.11**	0.70	0.47	-	0.93	5.37

**CD64**	**RA**	**0.89**	**0.78**	**-**	**1.00**	**76.31**	0.82	0.66	-	0.97	69.72
	RA(+)	0.66	0.00	-	1.00	65.72	0.63	0.00	-	1.00	63.09
	**RA(++)**	**1.00**	**1.00**	**-**	**1.00**	**100.00**	**0.95**	**0.78**	**-**	**1.00**	**88.39**

**CD90**	RA	0.83	0.69	-	0.97	57.97	0.78	0.62	-	0.94	53.55
	RA(+)	0.70	0.00	-	1.00	69.70	0.63	0.00	-	1.00	63.21
	RA(++)	0.78	0.59	-	0.98	10.12	0.72	0.50	-	0.94	6.58

**CD271**	RA	0.46	0.26	-	0.66	7.43	0.47	0.27	-	0.67	9.97
	RA(+)	0.03	0.03	-	0.03	3.31	0.03	0.03	-	0.03	3.28
	RA(++)	0.58	0.27	-	0.88	15.48	0.70	0.44	-	0.95	18.79

**CD304**	RA	0.72	0.54	-	0.89	42.98	0.69	0.50	-	0.87	42.23
	RA(+)	0.41	0.10	-	0.73	41.31	0.33	0.12	-	0.55	33.35
	**RA(++)**	0.82	0.62	-	1.00	45.95	**0.94**	**0.83**	**-**	**1.00**	**84.92**

**HLA-DR**	**RA**	**0.87**	**0.74**	**-**	**0.99**	**65.01**	**0.86**	**0.74**	**-**	**0.99**	**69.28**
	RA(+)	0.72	0.00	-	1.00	72.09	0.77	0.00	-	1.00	76.79
	RA(++)	0.82	0.59	-	1.00	53.56	0.77	0.49	-	1.00	55.01

Expression of listed surface antigens was analyzed on synovial tissue with LSC. Data were obtained with ROC analysis. Bold, AUC > 0.85.

### Classification of patient cohorts

Sensitivity values for classification based on expression of markers such as HLA-DR, CD90, CD29, or CD11b were in general higher for the affected area than for MFI, whereas specificity values vary among markers between affected area and MFI. However, multivariate discriminant analysis by combination of data for affected area and MFI within the same LDA/cross-validation run did not improve discriminatory capability. Cross-validation demonstrated that both MFI and the affected area are suitable for classification of RA as individual parameters. Hence, data for affected area and MFI were considered as discrete values for further analysis, but, for the sake of clarity, only results for MFI are further addressed. Cross-validation data are summarized in Additional file 1, Table S3.

**Figure 2:**
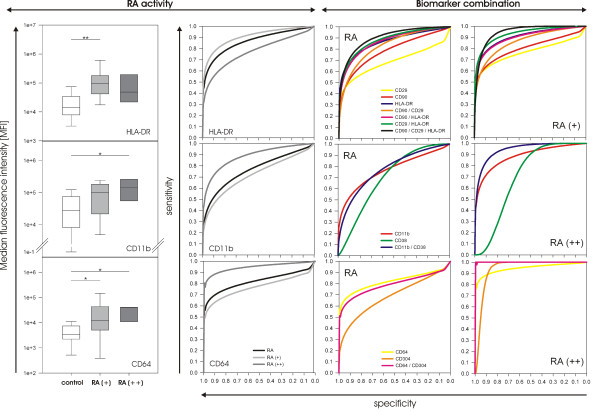
**Classification of rheumatoid arthritis (RA) subgroups and panel analysis**. Among the tested biomarker candidates, HLA-DR and CD64 had the highest discriminatory capability. However, most of the markers showed a clear preference for identifying one of both RA subgroups. Results are shown for median fluorescence intensity (MFI) values of RA(+) and RA(++). HLA-DR was a suitable marker for identification of RA(+) (*P *= 0.0006), whereas CD11b was better for classification of RA(++) (*P *= 0.0159). Expression of CD64 was significantly different from control for both RA(+) (*P *= 0.0156) and RA(++) (*P *= 0.004). Box plots show median and 25^th^/75^th ^percentile; whiskers, 5^th^/95^th ^percentile. Missing whiskers are due to the few RA(++) patients (*n *= 5). Higher sensitivity for the respective RA subgroup is also visible in receiver operating characteristic (ROC) analysis (center). Combination of markers increased sensitivity in classification of RA, as demonstrated by ROC analysis for MFI (right). Higher AUC values were obtained for the panels than for the individual markers (for AUC values, see Tables 2 and 3). However, the preference for one of both RA subgroups is obvious (last column). The same tendency is applied for analysis of affected area, although in this case, the increase did not reach the level of MFI. Confidence intervals in ROC graphs are not displayed because of visual simplicity.

Besides the ability to discriminate between RA and control, the expression of most of these markers depended on the status of current-activity synovitis. Whereas CD90, HLA-DR, CD64, and CD29 preferentially identified patients with mild current activity (RA(+)), CD64 and CD11b indicated high current activity and are therefore suitable markers to differentiate between RA(++) and control (Additional file 1, Table S2). CD11b has a higher sensitivity in classifying RA(++) than the whole RA cohort (Table 2, Figure 2). Besides CD11b and CD64, the plasmacytoid dendritic cell marker CD304 revealed the best discriminatory capability for control and RA(++), although it was not significant in the Mann-Whitney *U *test. With a sensitivity of 85%, it appeared to be a good marker for classification of RA(++) (Table 2). When compared with that of control individuals, CD64 was the only marker capable of discrimination between patients with mild and high current activity. Hence, CD64 seems not to be restricted to one subcohort of RA. However, it failed to discriminate between both states of activity (that is, between RA(+) and RA(++); see Additional file 1, Table S2).

### Combination of surface markers for improved RA classification

For improving the discriminatory capability, all possible two-mAb panels were tested *in silico *with a combination of measured data of individual biomarkers in LDA and cross-validation (Additional file 1, Table S4). Promising combinations were tested on serial sections from samples of all patients (Table 3).

**Table 3:** Discriminatory capability of biomarker panels

		Affected area					MFI					
				
Biomarker candidate	Control vs. cohort	AUC	95% Confidence interval			**Sensit. at 90% specif**.	AUC	95% Confidence interval			**Sensit. at 90% specif**.	
**CD38 and CD11b**	RA	0.77	0.61	-	0.94	46.82	0.77	0.61	-	0.94	44.95	
	RA(+)	0.69	0.48	-	0.89	28.45	0.70	0.49	-	0.90	29.12	
	**RA(++)**	**0.97**	**0.87**	**-**	**1.00**	**91.98**	**0.94**	**0.83**	**-**	**1.00**	**83.20**	

**CD304 and CD64**	RA	0.84	0.71	-	0.98	66.16	0.79	0.63	-	0.95	65.08	
	RA(+)	0.78	0.59	-	0.96	51.07	0.70	0.48	-	0.92	49.88	
	**RA(++)**	**1.00**	**1.00**	**-**	**1.00**	**100.00**	**1.00**	**1.00**	**-**	**1.00**	**100.00**	

**CD90 and CD29**	RA	0.81	0.67	-	0.96	56.42	0.83	0.68	-	0.97	55.84	
	**RA(+)**	**0.86**	**0.72**	**-**	**1.00**	**70.98**	**0.86**	**0.72**	**-**	**1.00**	**66.31**	
	RA(++)	0.67	0.42	-	0.92	9.24	0.75	0.49	-	1.00	35.48	

**HLA-DR and CD90**	**RA**	**0.87**	**0.74**	**-**	**0.99**	**65.46**	**0.87**	**0.74**	**-**	**0.99**	**66.53**	
	**RA(+)**	**0.89**	**0.75**	**-**	**1.00**	**73.92**	**0.90**	**0.77**	**-**	**1.00**	**75.96**	
	RA(++)	0.83	0.61	-	1.00	53.85	0.79	0.54	-	1.00	51.55	

**HLA-DR and CD29**	**RA**	**0.86**	**0.74**	**-**	**0.99**	**63.02**	**0.88**	**0.76**	**-**	**1.00**	**69.08**	
	**RA(+)**	**0.88**	**0.75**	**-**	**1.00**	**69.96**	**0.93**	**0.83**	**-**	**1.00**	**81.57**	
	RA(++)	0.84	0.66	-	1.00	44.26	0.76	0.49	-	1.00	47.12	

**CD29, CD90, and HLA-DR**	**RA**	**0.90**	**0.79**	**-**	**1.00**	**70.64**	**0.90**	**0.79**	**-**	**1.00**	**72.25**	
	**RA(+)**	**0.91**	**0.79**	**-**	**1.00**	**78.20**	**0.95**	**0.86**	**-**	**1.00**	**83.07**	
	**RA(++)**	**0.87**	**0.69**	**-**	**1.00**	**58.44**	0.71	0.46	-	0.97	56.13	

Listed panels of potential biomarkers were labeled simultaneously by the respective antibodies on synovial tissue and analyzed with LSC. Data were obtained with ROC analysis. **Bold**, AUC > 0.85.

In general, LSC analysis of panels provided similar values for sensitivity and specificity, as predicted by LDA/cross-validation. The best discriminatory capability among the tested two-mAb panels yielded the simultaneous identification of HLA-DR/CD29 with AUC = 0.88 and 69% sensitivity at 90% specificity. The combination of HLA-DR and CD90 was comparably good. If the mAb panel tagged all three markers (CD29, CD90, and HLA-DR), sensitivity increased up to 72%, and AUC = 0.90 (Table 3). For classification of RA(+), slightly better sensitivity and AUC values were obtained for the previously mentioned two-mAb panels and the three-mAb panel. The CD304/CD64 combination confirmed the expectations as a suitable biomarker set for RA(++) classification (AUC = 1). For discrimination of RA and control, this panel is applicable. The CD11b/CD38 panel reached a high accuracy in classifying RA(++), as well (AUC = 0.94; 83% sensitivity). The high sensitivity of these panels makes them an appropriate diagnostic tool for highly acute synovitis in RA (Table 3).

Figure 2 (right panel) very clearly shows the improved discrimination of RA and control when using mAb panels. The combination of CD29 and CD90 yielded higher AUC values than did both markers measured individually. When HLA-DR was added to the set of antibodies, the highest AUC value was obtained. As expected from the single-marker performance, the identification of mild current-activity synovitis (RA(+) patients) was even better when combining markers into panels (Table 3 and Figure 2, right panel). Hence, the simultaneous measurement of the expression of multiple proteins (biomarker panels) resulted in a better discrimination between RA (or RA subgroups with different states of current activity) and controls than did the determination of the expression of one surface protein alone.

## Discussion

Varying disease conditions are among the most challenging problems in RA diagnostics. In many patients, the symptomatic chronic inflammation results in bone and joint erosion. Early detection of inflammatory signs in RA is important and clearly shows a higher probability for successful treatment. For the last two decades, several studies have aimed to assess the degree of arthritis intensity with scintigraphic approaches. With techniques such as targeted immunoscintigraphy, the presence of relevant molecules can be highlighted by radiolabeled mAbs [4,19,20]. On the basis of the given amount of these relevant molecules involved in the pathophysiology of RA, it allows better staging of the disease and might provide a possibility to perform "evidence-based biologic therapy" for arthritis. However, a disadvantage of this method is mainly the intricacy of binding and biodistribution properties of new potential biomarkers. It is important that needed information on ligands cannot be evaluated, for safety reasons, in the patient first. In this study, we were able to identify potential biomarkers and biomarker panels of RA in the synovial tissue for future application in immunoscintigraphy, immuno-PET, or *in vivo *fluorescence imaging. Furthermore, we were able to show that LSC, a microscope-based cytometry technique, is a sophisticated tool for objective, unbiased, and quantitative validation of such biomarkers.

Although peripheral blood or synovial fluid can much more easily be taken from the patient than can tissue samples, biomarker identification by LSC and appropriate statistical test algorithms, such as ROC analysis, provide several advantages. First, tissue analysis directly reflects the *in vivo *situation in the affected joint. Second, automated high-content quantification of fluorochrome-labeled biomarkers by LSC offers time-efficient and unbiased data collection without interobserver variations. Moreover, the obtained parameters (MFI and affected area) quantified *in situ *with the presented monocolor analysis by LSC might allow a reliable prediction of the immunoscintigraphic outcome *in vivo*.

Diagnostic performance of biomarkers certainly depends on disease duration and current inflammatory and destructive processes. Even though we investigated synovial tissue of patients with long-standing RA, variations in disease occur. Hence, cell infiltration and thus biomarker expression may also vary. In this study, we used the grade of current activity in synovitis for subtyping the RA cohort. In contrast to the systemic inflammatory activity measured by ESR and CRP levels, the current activity provides information on exudative inflammatory processes on the synovial surface (that is, directly in the affected joint), in particular, fibrin exudation and granulocyte emigration [15]. We found differences in expression of investigated markers depending on the current-activity status of synovitis in long-standing RA disease. An increased expression of CD64, CD11b, and CD304 was highly specific for patients with a high current activity in synovitis, making them suitable markers to monitor active inflammation processes. An elevated expression of CD90 and CD29, conversely, is an indication of mild current activity in RA synovitis. Biomarkers identifying highly local inflammatory processes in synovial tissue might in future be used for radiodiagnostic approaches that would allow a differentiation of patients with active synovitis from those with disease remission. This was already proven for the radiolabeled anti-CD3 antibody [20].

The expression of CD64 (FcγRI), a prominent antigen on the surface of macrophages, was highly discriminative for RA in this study, as good as anti-cyclic citrullinated protein antibodies (anti-CCP) in other studies [21]. We found that an increased severity grade of current activity in synovitis leads to an elevated expression of CD64 in the synovial tissue. Hence, CD64 might be used as a sensitive biomarker to predict local inflammation and possible efficacy of antirheumatic treatment, as already proposed for CD68 [22]. However, CD64 might be more suitable for *in vivo *diagnostic techniques such as immunoscintigraphy or immuno-PET, as it is located on the cell surface, whereas CD68 is predominantly intracellularly expressed, which is relevant only for *in situ *diagnostic techniques [23].

In the majority of diseases, as in RA, diagnosis is based on a minimum of two or more essential physiological parameters [14]. As several cell types are involved in RA pathology, it is reasonable to identify at least two cell types or their activation states and combine them for analysis as a biomarker panel. In the present study, we demonstrated that the combined expression level of HLA-DR and CD90 or CD29 had a higher discriminatory capability than did the expression of the individual antigens alone. The combination of all three markers had a high sensitivity in classifying RA(+), making it an appropriate diagnostic panel for cases of RA with mild current synovitis. For identification of highly current synovitis, a panel including CD64 and the plasmacytoid dendritic cell marker CD304 or the combination of CD11b and CD38 was more suitable (Figure 2, right panel, and Table 3).

CD64 and HLA-DR, as well as the biomarker panels CD64/CD304 and HLA-DR/CD90/CD29, were found to be clearly discriminative between long-standing RA (or RA subgroups with mild or high current activity in synovitis) and acute non-RA arthritis. However, the use of identified biomarkers and biomarker panels for diagnosis of early RA and the expression of them in the synovial fluid should also be investigated. Moreover, a comprehensive study with the inclusion of further inflammatory arthritic joint diseases, such as osteoarthritis (OA) or reactive arthritis (ReA), should be initiated in the near future.

## Conclusion

In this study, we demonstrated that (a) the expression level of surface proteins on cells in the synovial tissue can be used for the discrimination for patients of RA and trauma-induced acute arthritis; (b) the expression level of most of the tested markers is influenced by the severity grade of current activity in synovitis, which implies their suitability as biomarkers for detection of inflammatory process in the joint; and (c) the measurement of a combined expression level of up to three surface proteins (biomarker panel) increases sensitivity.

The combination of HLA-DR/CD90/CD29 is a newly described promising biomarker panel, and the determination of its combined expression is reliable for the discrimination between RA and acute non-RA arthritis. The combination of CD64 and CD304 or CD11b with CD38 might be used as a novel biomarker panel to detect highly active synovitis in RA. The presented LSC-based analysis represents a promising approach for biomarker validation in RA.

## Abbreviations

ACR: American College of Rheumatology; AUC: area under the curve; EULAR European League Against Rheumatism; LDA: linear discriminant analysis; LSC: laser scanning cytometry; mAb: monoclonal antibody; MFI: median fluorescence intensity of the analyzed tissue; PET: positron-emission tomography; RA: rheumatoid arthritis; RA(+): subgroup of RA patients showing no or only mild signs of current-activity synovitis in the synovial tissue; RA(++): subgroup of RA patients with signs of high current-activity synovitis in the synovial tissue; ROC: receiver operating characteristic.

## Competing interests

The authors declare that they have no competing interests.

## Authors' contributions

CF and AM carried out the experimental selection, design, and performance, analyzed and interpreted the data, and drafted the manuscript. JK carried out the experimental design, interpreted the data, and drafted the manuscript. MB, PH, and RS carried out orthopedic surgery, sample acquisition and characterization, and helped to draft the manuscript and to interpret the data. UW, US, and FE participated in the project acquisition, design, and coordination, and helped to draft the manuscript. AT carried out the project design and coordination, experimental design, and manuscript preparation. JL conceived of the study, carried out the design and coordination of the project, the experimental design, and manuscript preparation. All authors read and approved the manuscript for publication.

## Supplementary Material

Additional file 1 **The supplementary information contains more-detailed sections of patients and methods (histology, staining procedures, fluorescence analysis, and data-acquisition strategy)**. In addition, four supplementary figures show the overall procedure of validation, immunofluorescence staining, LSC data-acquisition and analysis strategies. Figure s1. Principle of *in vitro *validation and selection of mAbs as specific biomarkers for RA. Figure s2. Immunofluorescence staining. Figure s3. LSC analysis. Figure s4. Tissue analysis. Supplementary Tables 1 through 4 include data on tested antibodies and additional results of cross-validation and multivariate data analysis. Table s1. Examined surface antigens. Table s2. Comparison between cohorts, considering RA subgroups. Table s3. Cross-validation. Table s4. Multivariate analysis. Click here for file
