# Supplementary material for: Identification and evaluation of novel synovial tissue biomarkers in rheumatoid arthritis by laser scanning cytometry
Source: Arthritis Res Ther. 2012 Jan 17;14(1):R8. doi: 10.1186/ar3682 (PMC3392796; doi:10.1186/ar3682)
Supplement: Additional file 1 — The supplementary information contains more-detailed sections of patients and methods (histology, staining procedures, fluorescence analysis, and data-acquisition strategy). In addition, four supplementary figures show the overall procedure of validation, immunofluorescence staining, LSC data-acquisition and analysis strategies. Figure s1. Principle of in vitro validation and selection of mAbs as specific biomarkers for RA. Figure s2. Immunofluorescence staining. Figure s3. LSC analysis. Figure s4. Tissue analysis. Supplementary Tables 1 through 4 include data on tested antibodies and additional results of cross-validation and multivariate data analysis. Table s1. Examined surface antigens. Table s2. Comparison between cohorts, considering RA subgroups. Table s3. Cross-validation. Table s4. Multivariate analysis. [file ar3682-S1.PDF]

Supplementary Information 1

Patients and Samples

Synovial tissue samples were obtained by joint surgery on 17 patients with long-term RA and 14 patients with non-RA arthritis.

After excision, samples were transferred immediately into phosphate-buffered saline (PBS) containing antibiotics (penicillin (1000µg/ml) and streptomycin (1000U/ml); both Biochrom, Berlin, Germany). Within 2 hours, synovial tissue samples were embedded in Tissue Tek® (Sakura, Heppenheim, Germany) and snap-frozen in liquid nitrogen. Deep-frozen blocks were stored at -80°C until sectioning. Cryosections (thickness: 6µm for fluorescence microscopy or 8µm for LSC) were prepared using a Cryocut (Leica Biosystems, Nussloch, Germany) and mounted on Superfrost Plus® adhesive glass slides (Menzel Glas, Braunschweig, Germany). Slides were stored at -80°C until further analysis.

Histology

Haematoxylin and Eosin (H&E) staining was performed on cryosections of each specimen according to standard protocols using a Leica Multistainer 5020 (Leica Microsystems, Wetzlar, Germany). Briefly, slides were placed for 30sec in distilled water followed by haematoxylin (Merck, Darmstadt, Germany) staining for 30sec. Subsequently, slides were washed with running tap water for 5min and then counterstained with eosin (0.5% in Ethanol; Sigma Aldrich, Taufkirchen, Germany) for 2min. Tissue sections were dehydrated in 70%/30% v/v ethanol/water and were then dipped into solutions of increasing volume fraction of ethanol (80% - 100% v/v) for 10sec per step and in pure isopropanol (Merck) for 1min. Finally, sections were incubated in xylene (Merck) for 2min and covered with Entellan® (Merck).

Slides were analysed using a brightfield/fluorescence microscope (Axio Imager; Zeiss, Jena, Germany). H&E-stained synovium samples were surveyed by a pathologist. Visual inspection provided information on cellular architecture and number of infiltrating cells. Synovial samples from RA patients as well as those from the control group showed moderate to marked synovial cell hyperplasia, vascular proliferation and infiltration of mononuclear cells. The intensity of mononuclear cell infiltration and sub-synovial lining varied greatly among individual patients. Additionally, synovial sections were classified by the grade of current activity resulting in the RA sub-groups RA (+) and RA (++).

Fluorescence analysis

Selection of appropriate antibodies

Series of cryosections of synovium from eight RA patients were stained with mAbs (Table s1) labelled with fluorescein isothiocyanate (FITC), phycoerythrin (PE), or allophycocyanin (APC). Fluorescence images of the stained synovial sections were analysed by a brightfield/fluorescence microscope (Axio Vision, Zeiss) (Figure s2). Samples were surveyed investigator-blinded by two persons and scored on a scale from 0 to 3 indicating absent (0), mild (1), moderate (2), or marked (3) expression. Surface proteins with a minimal expression of moderate to marked were considered for further quantitative analysis by LSC (Table s1 upper part).

Mouse IgG1 labelled with FITC (1:100), APC (1:20) or PE (1:20) (all Beckman Coulter) was used as isotype control.

For indirect labelling, anti-CD90 (1:40) and anti-HLA-DR (8 µg/ml) were used. PE-conjugated goat anti-mouse IgG (ab7002; 1:100; Abcam, Cambridge, UK) was used as secondary antibody and served as negative control as well.

Antibodies for LSC analysis

Preliminary tests yielded comparable results in LSC analysis of serial sections of synovial tissue stained with the same mAb but different fluorochromes (anti-HLA-DR-FITC, anti-HLA-DR-PE, anti-HLA-DR-APC; Beckman Coulter, Krefeld, Germany, clone: immu-357). Hence, combinations of mAbs and fluorochromes were selected based on availability.

Immunofluorescence staining was performed either with directly labelled mAbs or alternatively with unlabelled primary mouse mAbs and fluorochrome-conjugated secondary rat anti-mouse IgG1 following titration. The following directly labelled mAbs were used: anti-CD11b-FITC (1:100), anti-CD4-FITC (1:100), anti-CD38-FITC (1:100), anti-CD29-PE (1:150), anti-CD304-APC (1:20), anti-CD271-APC (1:20), and anti CD64-APC (1:20). Mouse IgG1 labelled with FITC (1:100), or APC (1:20) (both Beckman Coulter) was used as isotype control.

For indirect labelling, anti-CD90 (1:40) and anti-HLA-DR (8 µg/ml) were used. PE-conjugated goat anti-mouse IgG (ab7002; 1:100; Abcam, Cambridge, UK) was used as secondary antibody and served as negative control as well.

Antibody combinations

Linear discriminant analysis with subsequent cross-validation was performed with results obtained by LSC analysis of single labelled tissue sections. Based on the discriminatory capability of analysed markers, combinations of two and three markers were selected in order to increase discriminatory capability. The selected marker combinations (see results for details) were stained on serial sections for each patient using the same fluorochrome, i.e. FITC, PE, or APC respectively for all used markers at the same time. In order to minimise intra-assay variations, panels containing the same mAbs (including control samples) were stained in the same run for each patient.

Staining

Prior to staining, cryosections were thawed, dried and fixed in ice-cold 100 % acetone for 10min. Slides were immediately dried and quickly warmed to room temperature. Samples were pre-incubated with normal goat serum (10% in PBS) for 30min to block non-specific binding.

Direct staining

After blocking, samples were washed once with PBS containing 1% bovine serum albumin (BSA; Sigma Aldrich). For single staining, mAbs were diluted in PBS (1% BSA). Antibodies were mixed for double and triple labelling and diluted in PBS (1% BSA) with the same final concentrations as for single labelling. 150µl of this solution was added to a section and incubated for 90min at 20°C. Sections were rinsed 4 times with PBS (1% BSA) followed by two times washing in 0.05M TRIS buffer. Only sections used for microscopic analyses were additionally stained for DNA. To this end, sections were incubated with PBS containing 1µg/ml 4',6-diamidino-2-phenylindole (DAPI; Applichem, Darmstadt, Germany). After staining, all samples were mounted with anti-fade medium (Dako, Hamburg, Germany), covered with a cover slip and stored at 4°C until analysis.

Indirect staining:

Staining of primary mAbs was done as described for directly labelled mAbs. After incubation, sections were washed 4 times with PBS (1% BSA) followed by incubation with PE-conjugated secondary antibody for 1hr at 20°C. Afterwards, samples were rinsed (PBS / 1% BSA and TRIS), stained for DNA (only for microscopy) and mounted with medium as described above.

Data acquisition

Quantitative analysis of synovial tissue was performed by LSC. Settings for phantoms (equal-sized circles) were 10μm diameter, 10μm distance between the centres of phantoms and lattice pattern. In that way, the whole tissue was covered with roughly cell-sized contours in which fluorescence data were recorded for the respective fluorochrome in addition to the scatter signal of the tissue (Figure s3V).

The 488nm laser excitation line of the LSC was used for analysis of FITC and PE, whereas the 633nm Helium-Neon-laser was used for APC. Fluorescence detection was done by Photomultiplier tubes (PMTs) and appropriate bandpass filters. PMT voltages were set for non-saturated conditions. Optimised settings were applied for all measurements of the same fluorochrome.

SUPPLEMENTARY FIGURES

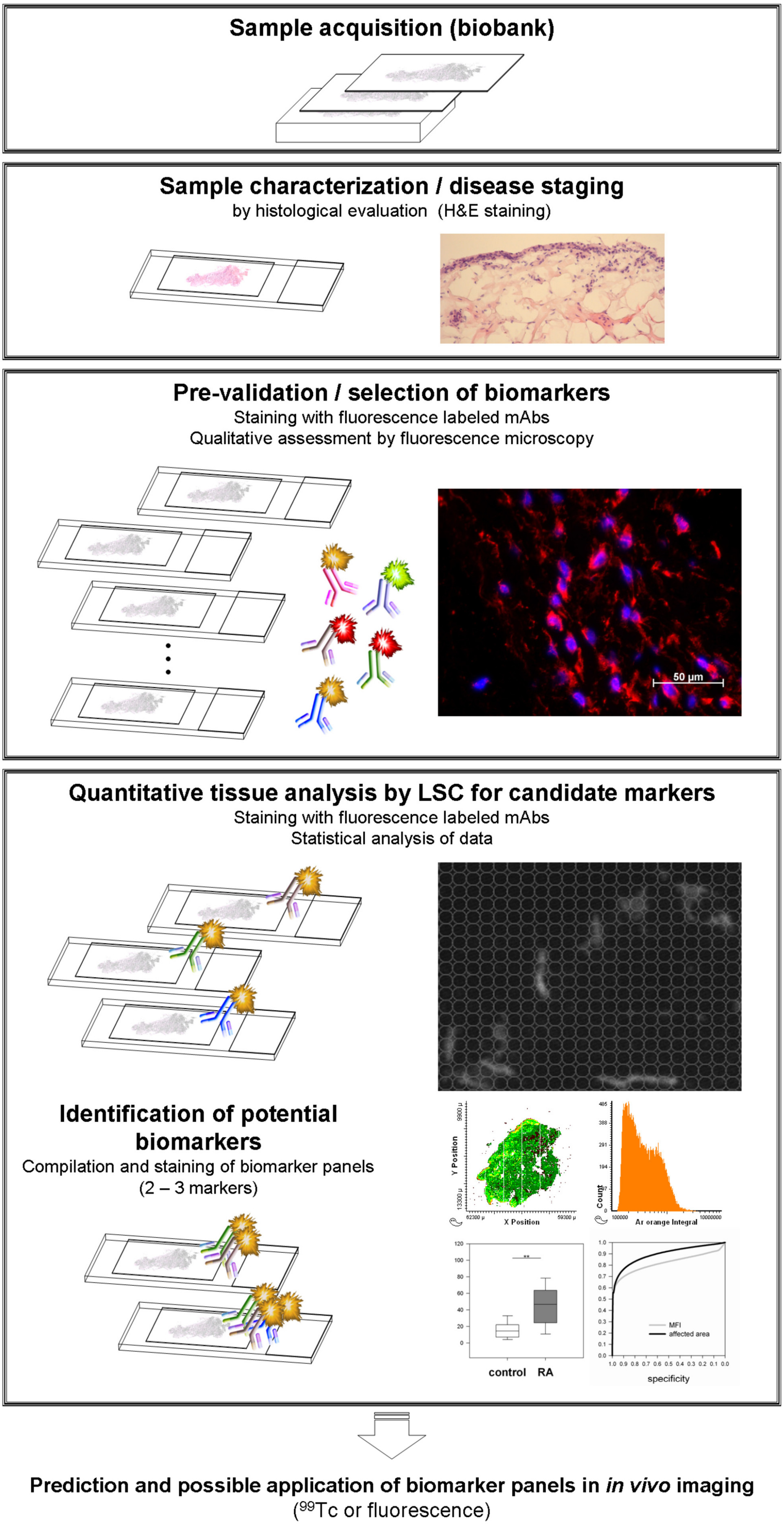

Figure s1: Principle of *in vitro* validation and selection of mAbs as specific biomarkers for RA. Quantitative LSC analysis allows prediction of binding and emulation of the *in vivo* imaging situation (immuno-scintigraphy, PET, or *in vivo* fluorescence imaging).

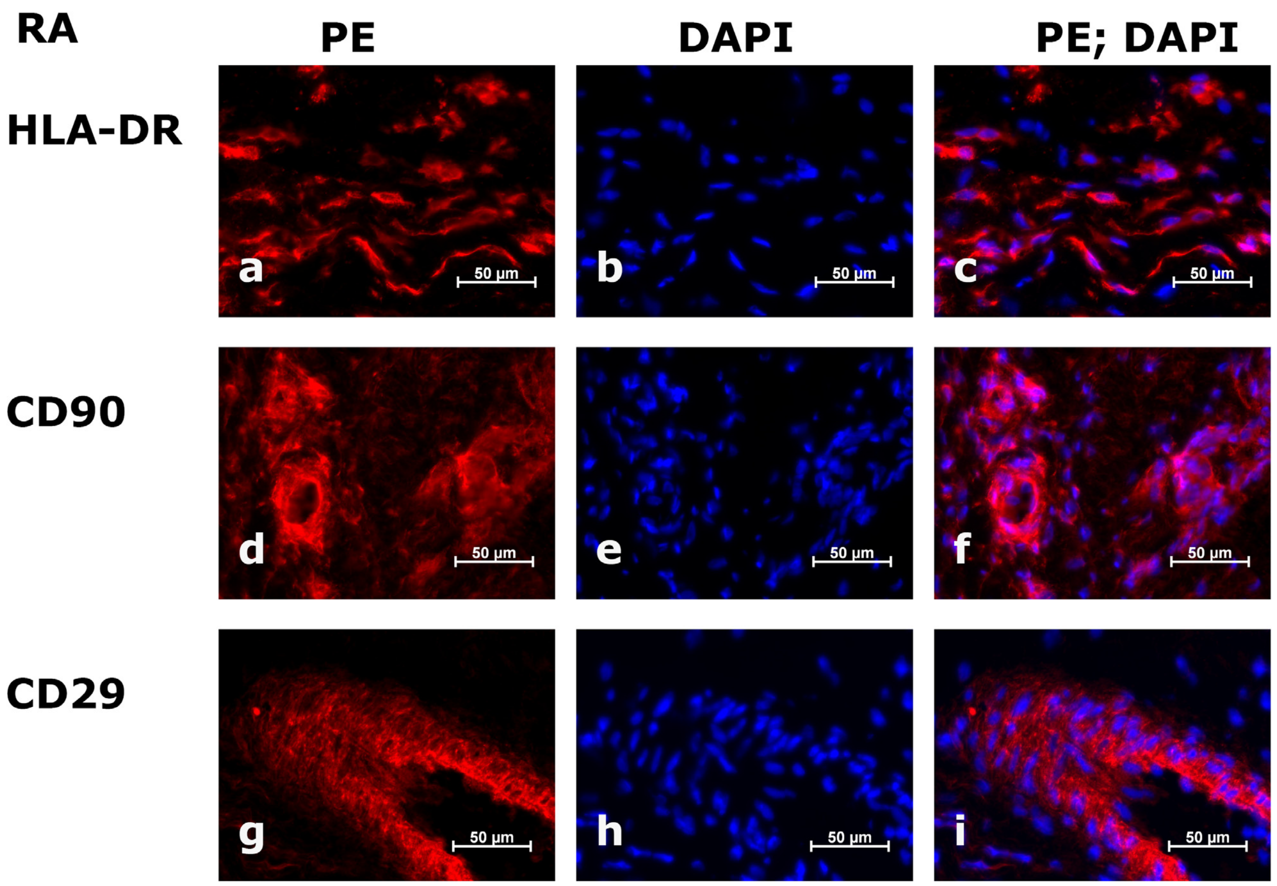

Figure s2: Immunofluorescence staining. For identification of surface markers, which could be suitable as biomarkers, cryosections of synovial tissue of RA (a-g) were labelled with anti-HLA-DR; anti-CD90; anti-CD29 conjugated with PE, DNA was counterstained with 4‘,6-diamidino-2-phenylindole (DAPI) (b;e;h) and merge (c;f;i;).Magnifications x400.

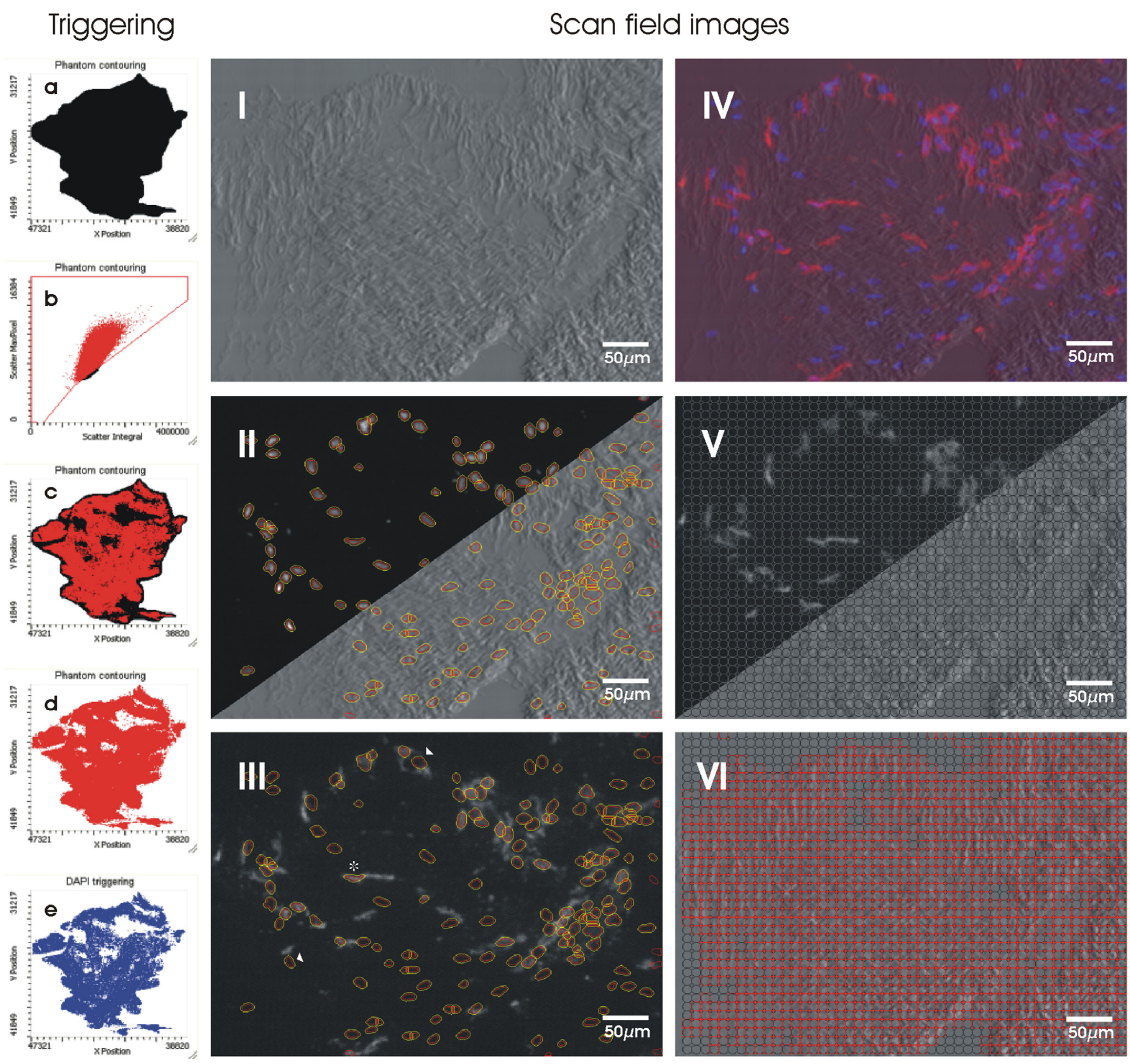

Figure s3: LSC analysis. Synovial membranes (8µm cryosections) from RA and non-RA arthritis patients were analysed for several potential biomarkers, exemplarily displayed for HLA-DR in this figure. Images I-VI show one example scan field of LSC analysis; an overlay image of all relevant channels is shown in IV (scatter: grey, DAPI: blue, HLA-DR red). Tissue on the slide is visible by its scatter signal (I). Triggering on DAPI signal (II upper part: DAPI signal with trigger contour) has been abandoned in favour of phantom contouring (V). As DAPI is restricted to DNA, only regions of nuclear presence would be included for further analysis but not the entire tissue, including cytoplasmic regions and intercellular tissue (II lower part: DAPI trigger contour applied to scatter channel). Furthermore, DNA triggering can lead to loss of information (i.e. of additional cellular staining; III: HLA-DR signal with applied DAPI trigger) in case of eccentric nuclei (arrow heads) or very long stretched cells (asterisk). For that reason, phantom contouring with phantom size similar to cell size was chosen since it covers the whole scan field, i.e. tissue (V: phantom contouring applied for HLA-DR (upper part) and scatter channel (lower part)) and no fluorescence information is lost. However, scan fields might contain also regions without tissue. This must be considered for analysis. Dot plot a) represents location of each phantom from LSC analysis without discrimination of tissue and tissue-free area,

i.e. displaying the entire scan area covered with phantoms. Scatter signal can be used for selection of phantoms covering tissue (b). Phantoms defined as tissue are marked in red (b-d and VI). Only those phantoms were used for further analysis (Figure s4). An example scan of the same tissue section with trigger set on DAPI is shown in e); the same shape of the tissue is visible indicating proper exclusion of non-tissue related phantoms in d).

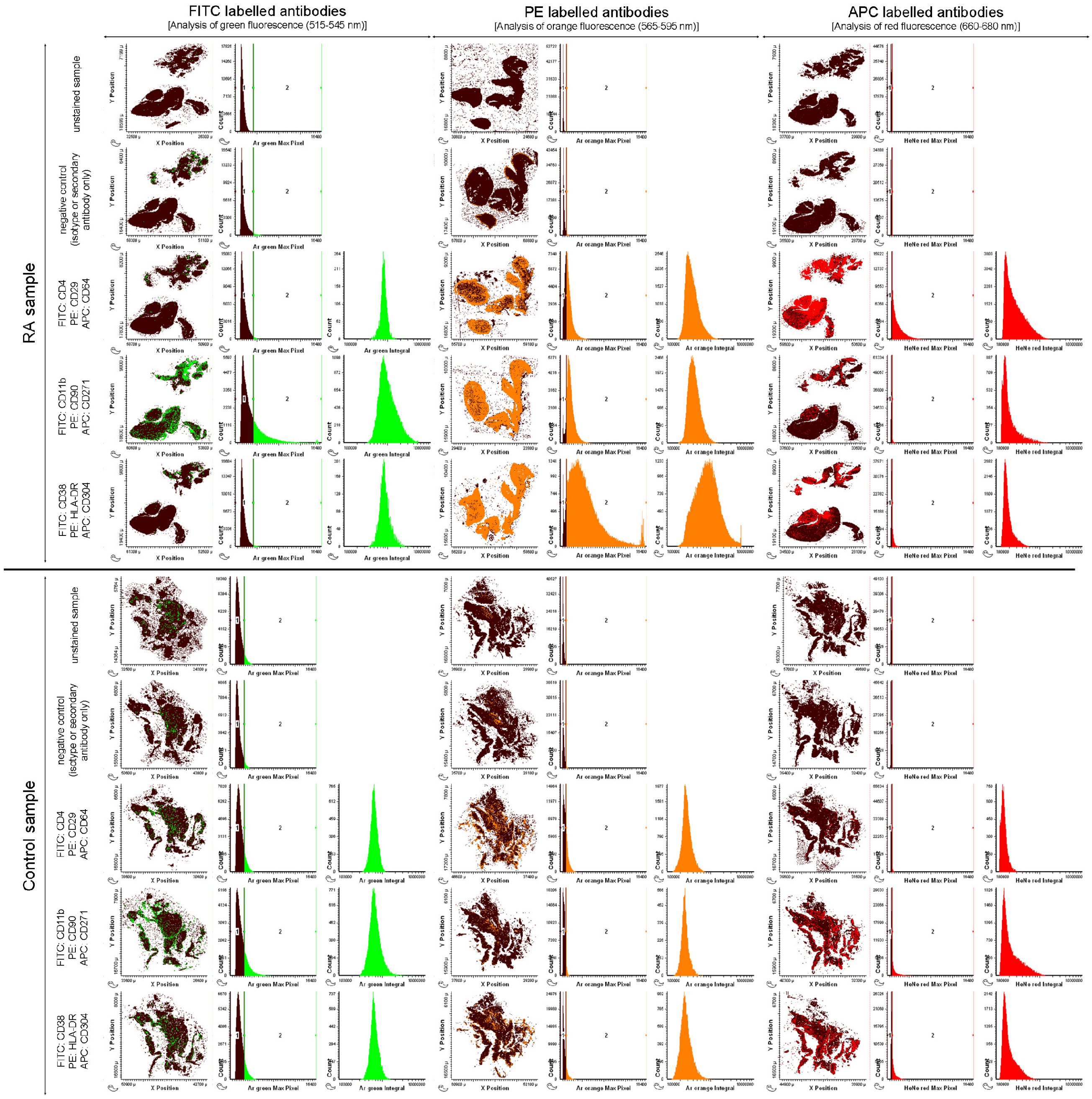

Figure s4: Tissue analysis. This figure shows the analysis of single stained tissue sections exemplarily on one RA sample and one control. The analysis of mAb panels was done the same way. Negative controls were analysed for each patient and with the same settings than for tissue sections with antibodies. In order to determine the area of tissue being positive for the used marker, the parameter MaxPixel was used. Based on isotype control measurements (secondary antibody controls in case of PE staining), the threshold (<5% positive events) for autofluorescence was set for each fluorescence dye and patient, respectively (column 2, 5, 8, first row for both shown samples). For each analysis the percentage of phantoms with a MaxPixel value above this threshold (affected area) was determined (column 2, 5, 8). MFI values were obtained from median integral values of all tissue-related phantoms (column 3, 6, 9). MFI values were corrected by subtraction of the respective MFI negative control value.

	mAb	Score	clone	distributor
LSC analysis	CD11b	3	BEAR1	Beckman Coulter, Krefeld, Germany
	CD38	3	T16	Beckman Coulter, Krefeld, Germany
	CD29	3	4B4LDC9LDH	Beckman Coulter, Krefeld, Germany
	CD90	3	AS02	Laboratory, Dr. A. Saalbach *
	HLA-DR	3	BL-HLA-DR	DiaMak, Leipzig, Germany (purified in the IZI laboratory)
	CD64	3	10,1	biozol, Munich, Germany
	CD304	3	AD5-17F6	Miltenyi Biotec, Bergisch-Gladbach, Germany
	CD4	2-3	13B8.2	Beckman Coulter, Krefeld, Germany
	CD271	2-3	ME20.4-1H4	Miltenyi Biotec, Bergisch-Gladbach, Germany
	CD14	2	M5E2	BD Pharmingen, Heidelberg, Germany
	CD16	2	3G8	Beckman Coulter, Krefeld, Germany
	CD1a	1	H1149	BD Pharmingen, Heidelberg, Germany
	CD3	1	UCHT1	Beckman Coulter, Krefeld, Germany
	CD40	1	mAb89	Immunotech, Marseille, France
	CD45	1	J33	Beckman Coulter, Krefeld, Germany
	CD45RO	1	BL-45RO	DiaMak, Leipzig, Germany
	CD83	1	HB15A	IQ Products, Groningen, The Netherlands
	CD86	1	B-T7	IQ Products, Groningen, The Netherlands
	CD25	0	BC96	eBioscience, Hatfield, UK
	CD28	0	CD28.2	eBioscience, Hatfield, UK
	CD49e	0	SAM1	Beckman Coulter, Krefeld, Germany
	CD50	0	MEM-171	Immunotools, Friesoythe, Germany
	CD54	0	84H10	Immunotech, Marseille, France
	CD62L	0	IM2655	Beckman Coulter, Krefeld, Germany
	CD62P	0	CLB-Thromb/6	Immunotech, Marseille, France
	CD69	0	L78	BD Biosciences, Heidelberg, Germany
	CD80	0	MAB104	Beckman Coulter, Krefeld, Germany
	CD152	0	BNI3	Immunotech, Marseille, France

* Gift from Dr. A. Saalbach, Dept. of Dermatology, University Hospital Leipzig, Germany

Table s1: Examined surface antigens. Several antibodies directed against cell surface antigens were tested on cryosections of synovium. Expression of the listed markers was surveyed blindly by two persons on a fluorescence microscope and scored as follows: absent (0), mild (1), moderate (2), and marked (3). Fluorescence labelled antibodies with low unspecific binding and clearly visible specific fluorescence signals (i.e. revealing at least moderate to marked protein expression) were taken for LSC analysis. *Gift from Dr. A. Saalbach, Dept. of Dermatology, University Hospital Leipzig, Germany

	biomarker candidate / biomarker panel	ANOVA	Mann-Whitney *U* test			
			control samples vs.			RA (+) vs. RA (++) *
			RA	RA (+) *	RA (++) *	
affected area	**CD4**	0.37941	0.19689	0.30346	0.24695	0.52709
	**CD11b**	**0.01246**	**0.00880**	0.07183	**0.00410**	0.20590
	**CD29**	**0.00847**	**0.00255**	**0.00467**	0.05187	0.29184
	**CD38**	0.06723	0.09545	0.32836	0.03322	0.07296
	**CD64**	**0.00056**	**0.00026**	**0.00337**	**0.00119**	0.14002
	**CD90**	**0.00448**	**0.00196**	**0.00241**	0.07857	0.11385
	**CD271**	0.60114	0.55153	0.41054	0.92624	0.36997
	**CD304**	0.06331	**0.02902**	0.08963	0.04167	0.34278
	**HLA-DR**	**0.00302**	**0.00074**	**0.00100**	0.05187	0.67329
	**CD38 & CD11b**	**0.00507**	**0.01546**	0.14982	**0.00225**	0.02039
	**CD304 & CD64**	**0.00215**	**0.00171**	0.02363	**0.00119**	0.11385
	**CD90 & CD29**	**0.00452**	**0.00376**	**0.00203**	0.22876	0.05778
	**HLA-DR & CD90**	**0.00241**	**0.00074**	**0.00119**	0.04167	0.24625
	**HLA-DR & CD29**	**0.00228**	**0.00064**	**0.00119**	0.03322	0.29184
	**CD29 & CD90 & HLA-DR**	**0.00073**	**0.00022**	**0.00047**	0.02064	0.17059
MFI	**CD4**	0.77899	0.53147	0.71313	0.42411	0.83098
	**CD11b**	**0.03169**	**0.01622**	0.07548	**0.01589**	0.29184
	**CD29**	**0.02274**	0.07403	**0.01563**	0.85310	0.03060
	**CD38**	0.24025	0.11950	0.21261	0.15838	0.39908
	**CD64**	**0.00533**	**0.00196**	**0.01563**	**0.00410**	0.39908
	**CD90**	**0.00619**	**0.00426**	**0.00337**	0.16491	0.05778
	**CD271**	0.23995	0.82716	0.39599	0.30849	0.09169
	**CD304**	**0.02822**	0.07088	0.42524	**0.00548**	0.07314
	**HLA-DR**	**0.00264**	**0.00074**	**0.00057**	0.09562	0.59816
	**CD38 & CD11b**	**0.00833**	**0.01104**	0.08941	**0.00409**	0.05778
	**CD304 & CD64**	**0.00405**	**0.00695**	0.08963	**0.00119**	0.05778
	**CD90 & CD29**	**0.00556**	**0.00291**	**0.00241**	0.13852	0.11385
	**HLA-DR & CD90**	**0.00194**	**0.00074**	**0.00069**	0.07857	0.20590
	**HLA-DR & CD29**	**0.00106**	**0.00048**	**0.00021**	0.13852	0.24625
	**CD29 & CD90 & HLA-DR**	**0.00046**	**0.00019**	**0.00014**	0.06408	0.17059

Table s2: Comparison between cohorts considering RA sub-groups. Kruskal-Wallis one-way ANOVA was applied for comparison of control and the RA subgroups RA (+) and RA (++). Bold numbers indicate significant differences ($p \leq 0.05$) between groups. Mann-Whitney *U* test was used for detailed group comparison. Markers found to be significant for the entire RA group (second column) were mainly significant for one of the RA sub-groups only. CD64 is the only tested marker with significant differences of both RA subgroups to control. However, all markers failed to distinguish between RA (+) and RA (++). *Significance level was adjusted by Bonferroni correction in case of multivariate analyses ($p \leq 0.017$).

	biomarker candidate / biomarker panel	sensitivity	specificity	accuracy	likelihood ratio
affected area	**CD4**	74.23	10.95	45.33	0.83
	**CD11b**	72.39	67.88	70.33	2.25
	**CD29**	76.69	67.88	72.67	2.39
	**CD38**	56.44	51.09	54.00	1.15
	**CD64**	71.17	92.70	81.00	9.75
	**CD90**	66.26	70.80	68.33	2.27
	**CD271**	73.62	0.00	40.00	0.74
	**CD304**	71.17	56.20	64.33	1.62
	**HLA-DR**	85.28	78.10	82.00	3.89
	**CD38 & CD11b**	65.64	64.23	65.00	1.84
	**CD304 & CD64**	75.46	62.04	69.33	1.99
	**CD90 & CD29**	75.46	62.77	69.67	2.03
	**HLA-DR & CD90**	77.30	78.10	77.67	3.53
	**HLA-DR & CD29**	73.01	69.34	71.33	2.38
	**CD29 & CD90 & HLA-DR**	82.82	72.99	78.33	3.07
MFI	**CD4**	74.85	2.92	42.00	0.77
	**CD11b**	65.03	72.99	68.67	2.41
	**CD29**	55.21	57.66	56.33	1.30
	**CD38**	59.51	42.34	51.67	1.03
	**CD64**	59.51	70.80	64.67	2.04
	**CD90**	53.99	79.56	65.67	2.64
	**CD271**	79.14	0.00	43.00	0.79
	**CD304**	58.90	59.12	59.00	1.44
	**HLA-DR**	61.96	81.02	70.67	3.26
	**CD38 & CD11b**	64.42	72.26	68.67	2.32
	**CD304 & CD64**	59.51	73.72	59.33	2.26
	**CD90 & CD29**	60.12	80.29	64.67	3.05
	**HLA-DR & CD90**	60.12	88.32	64.00	5.15
	**HLA-DR & CD29**	65.64	90.51	69.33	6.92
	**CD29 & CD90 & HLA-DR**	61.96	94.16	62.27	10.61

Table s3: Cross-validation. Results are shown for all tested biomarkers and biomarker panels. Sensitivity and specificity values are listed for discrimination of RA and control.

	affected area			MFI		
**biomarker panel**	**sensitivity**	**specificity**	**accuracy**	**sensitivity**	**specificity**	**accuracy**
**CD11b & CD4**	74.23	62.04	68.67	64.42	69.34	66.67
**CD38 & CD4**	51.53	51.09	51.33	57.06	36.50	47.67
**CD29 & CD4**	75.46	64.96	70.67	53.99	51.82	53.00
**CD90 & CD4**	65.03	72.26	68.33	49.69	76.64	62.00
**HLA-DR & CD4**	84.66	78.10	81.67	54.60	77.37	65.00
**CD64 & CD4**	71.17	92.70	81.00	52.76	58.39	55.33
**CD271 & CD4**	62.58	5.84	36.67	65.64	4.38	37.67
**CD304 & CD4**	69.33	50.36	60.67	52.15	51.82	52.00
**CD38 & CD11b**	65.03	59.85	62.67	65.03	72.99	68.67
**CD29 & CD11b**	70.55	70.80	70.67	61.35	72.26	66.33
**CD90 & CD11b**	72.39	78.10	75.00	68.71	83.21	75.33
**HLA-DR & CD11b**	83.44	78.10	81.00	58.28	79.56	68.00
**CD64 & CD11b**	70.55	92.70	80.67	66.87	75.91	71.00
**CD271 & CD11b**	66.26	70.07	68.00	65.03	62.77	64.00
**CD304 & CD11b**	76.69	64.23	71.00	67.48	62.77	65.33
**CD29 & CD38**	77.91	65.69	72.33	59.51	54.01	57.00
**CD90 & CD38**	76.07	73.72	75.00	60.12	81.75	70.00
**HLA-DR & CD38**	83.44	78.10	81.00	66.26	86.13	75.33
**CD64 & CD38**	71.17	91.97	80.67	53.37	66.42	59.33
**CD271 & CD38**	56.44	50.36	53.67	52.76	35.77	45.00
**CD304 & CD38**	69.33	57.66	64.00	55.21	61.31	58.00
**CD90 & CD29**	67.48	70.80	69.00	55.83	75.18	64.67
**HLA-DR & CD29**	83.44	78.10	81.00	54.60	75.18	64.00
**CD64 & CD29**	80.98	87.59	84.00	64.42	77.37	70.33
**CD271 & CD29**	69.33	64.96	67.33	55.21	57.66	56.33
**CD304 & CD29**	82.21	70.07	76.67	57.06	74.45	65.00
**HLA-DR & CD90**	87.73	78.10	83.33	59.51	81.02	69.33
**CD64 & CD90**	88.96	83.94	86.67	65.64	86.13	75.00
**CD271 & CD90**	64.42	70.80	67.33	53.99	78.10	65.00
**CD304 & CD90**	80.37	72.99	77.00	65.03	81.02	72.33
**CD64 & HLA-DR**	87.73	78.10	83.33	65.64	94.89	79.00
**CD271 & HLA-DR**	76.69	78.10	77.33	61.96	80.29	70.33
**CD304 & HLA-DR**	88.96	78.10	84.00	62.58	83.21	72.00
**CD271 & CD64**	60.12	92.70	75.00	52.76	65.69	58.67
**CD304 & CD64**	69.33	92.70	80.00	50.92	69.34	59.33
**CD304 & CD271**	66.87	64.96	66.00	57.06	54.01	55.67

Table s4: Multivariate analysis. The table summarises the cross-validation results obtained from the combination of affected area (or MFI) for two individual biomarkers.
